# Novel 1q22-q23.1 duplication in a patient with lambdoid and metopic craniosynostosis, muscular hypotonia, and psychomotor retardation

**DOI:** 10.1007/s13353-018-0447-4

**Published:** 2018-05-29

**Authors:** Anna Sowińska-Seidler, Ewelina M. Olech, Magdalena Socha, Dawid Larysz, Aleksander Jamsheer

**Affiliations:** 1 Department of Medical Genetics, Poznan University of Medical Sciences, Rokietnicka 8 Street, 60-806 Poznan, Poland; 2 Department of Radiotherapy, The Maria Skłodowska Curie Memorial Cancer Centre and Institute of Oncology, Gliwice Branch, 44-101 Gliwice, Poland

**Keywords:** Craniosynostosis, Psychomotor retardation, 1q22-q23.1 duplication, LMNA, BGLAP

## Abstract

Craniosynostosis (CS) refers to the group of craniofacial malformations characterized by the premature closure of one or more cranial sutures. The disorder is clinically and genetically heterogeneous and occurs usually as an isolated trait, but can also be syndromic. In 30–60% of patients, CS is caused by known genetic factors; however, in the rest of the cases, causative molecular lesions remain unknown. In this paper, we report on a sporadic male patient affected by complex CS (metopic and unilateral lambdoid synostosis), muscular hypotonia, psychomotor retardation, and facial dysmorphism. Since a subset of CS results from submicroscopic chromosomal aberrations, we performed array comparative genomic hybridization (array CGH) in order to identify possibly causative copy-number variation. Array CGH followed by breakpoint sequencing revealed a previously unreported de novo 1.26 Mb duplication at chromosome 1q22-q23.1 that encompassed two genes involved in osteoblast differentiation: *BGLAP*, encoding osteocalcin (OCN), and *LMNA*, encoding lamin A/C. OCN is a major component of bone extracellular matrix and a marker of osteogenesis, whereas mutations in *LMNA* cause several genetic disorders called laminopathies, including mandibuloacral dysostosis (MAD) that manifests with low bone mass, severe bone deformities, and delayed closure of the cranial sutures. Since *LMNA* and *BGLAP* overexpression promote osteoblast differentiation and calcification, phenotype of our patient may result from misexpression of the genes. Based on our findings, we hypothesize that both *LMNA* and *BGLAP* may be implicated in the pathogenesis of CS in humans. However, further studies are needed to establish the exact pathomechanism underlying development of this defect.

## Introduction

Craniosynostosis (CS), the premature fusion of the cranial sutures, occurs in about 1:2000 to 1:2500 live born infants and represents one of the most common congenital craniofacial malformation (Cohen 1979; French et al. 1990; Lajeunie et al. 1995). Premature fusion of one or more sutures leads to the distortions of the skull shape and early closure of the fontanelles, which in approximately 50% of cases results in raised intracranial pressure. Consequently, increased risk for cortex lesion with intellectual disability or visual and hearing impairment is observed (Renier et al. 1982; Morriss-Kay and Wilkie 2005).

CS is a clinically and genetically heterogeneous group of disorders that may either occur in rare syndromic forms or, more frequently, as nonsyndromic isolated trait (Wilkie et al. 2007). The vast majority of CS patients are sporadic; however, 10–15% cases show familial recurrence (Cohen 2002). Syndromic forms comprise over 180 different genetic conditions, in which CS is associated with a broad spectrum of clinical symptoms, including skeletal defects, digital malformations, facial dysmorphism, cardiac and genitourinary defects or other organ abnormalities (Gleeson et al. 2006; OMIM). The most commonly recognized CS syndromes that have well-defined clinical phenotype include Apert (MIM 101200), Crouzon (MIM 123500), Pfeiffer (MIM 101600), Muenke (MIM 602849), Saethre-Chotzen (MIM 101400), Jackson-Weiss (MIM 123150), and Antley-Bixler (MIM 207410) syndromes (Kutkowska-Kazmierczak et al. 2018). The underlying genetic cause of these disorders involves mutations in the *FGFR1*, *FGFR2*, *FGFR3*, and *TWIST1* genes (OMIM). Other less frequent disorders result from different mutations in the *TCF12*, *EFNB1*, *MSX2*, *ALX3*, *GLI3*, *IL11RA*, and *ERF* genes (Jabs et al. 1993; Twigg et al. 2009, 2013; Hurst et al. 2011; Keupp et al. 2013; Sharma et al. 2013; Kutkowska-Kazmierczak et al. 2018). Conversely, little is known about genetic etiology of isolated CS and in the majority of cases the underlying molecular defect remains unidentified. Nonetheless, a couple of studies have demonstrated that complex forms of the disease result from chromosomal microaberrations referred to as copy number variations (CNVs), which may account for up to 10–15% of CS (Stratton et al. 1986; Eshel et al. 2002; Wilkie et al. 2010; Massalska et al. 2014). Lambdoid CS, accounting only for 2–4% of the cases, represents its rarest form with entirely unknown molecular origin. The pathomechanism of CNVs could be explained by either gene dosage effect leading to overexpression or haploinsufficiency of a gene/genes or by cis-regulatory effect that leads to misexpression of a target gene resulting from change of its regulatory landscape (Klopocki et al. 2011).

In this paper, we describe a sporadic male proband affected by complex CS, composed of metopic and lambdoid synostosis, muscular hypotonia, psychomotor retardation, and facial dysmorphism, resulting from a previously unreported de novo 1.26 Mb duplication at chromosome 1q22-q23.1, encompassing two genes involved in osteoblastogenesis: *BGLAP* encoding osteocalcin (OCN) and *LMNA* encoding lamin A/C. To our knowledge, this is the first genetic abnormality found in a patient presenting with lambdoid CS and the first report concerning the putative contribution of a CNV affecting *BGLAP* and *LMNA* genes to the premature closure of the cranial sutures.

## Methods

### Array comparative genomic hybridization (array CGH)

Genomic DNA of the index patient and his parents was extracted from peripheral blood leukocytes using standard protocols. Array comparative genomic hybridization (array CGH) was performed with the use of high resolution 1.4 M NimbleGen oligonucleotide array CGH (*Roche NimbleGen*) according to standard protocols provided by the manufacturers. Analysis was carried out with Deva software (*Roche NimbleGen*). Analysis settings were as follows: aberration algorithm, ADM-2; threshold, 6.0; window size, 0.2 Mb; filter, five probes, log2ratio = 0.29. The genomic profile was visualized by the SignalMap software (*NimbleGen Systems Inc.*). The aberrant genomic locus was visualized and analyzed in Cytoscape v3.3.0 (Shannon et al. 2003).

### Quantitative real-time PCR (qPCR)

We performed a quantitative real-time PCR (qPCR) in the index patient and his parents in order to confirm array CGH results and to show the inheritance pattern of the identified duplication using ViiA™ 7 Real-Time thermal cycler (*Applied Biosystems*). The qPCR assay was designed to determine the number of copies within the 1q22-q23.1 locus. Three primer pairs were used to amplify the region of duplication and two primer pairs for the 5′ and 3′ flanking regions. Moreover, we used qPCR assay to narrow down the duplication region prior to breakpoint sequencing. This was carried out with the set of eight primer pairs. All reactions were run in triplicate. The results were normalized to albumin (*ALB*) and the copy number in each of the analyzed regions was determined by means of comparative DDCt method using healthy control DNA as a calibrator. In order to assure reliability of the assay, we performed sex determination of samples in reference to factor VIII (*F8*) located on X chromosome. Reaction conditions and primer sequences are available upon request.

### Breakpoint sequencing

The exact breakpoints of the rearrangement were determined with the use of polymerase chain reaction (PCR) with primers designed to amplify the DNA fragment spanning the 3′ and 5′ ends of the duplication at chromosome 1q22-q23.1. Sequencing of the PCR product was carried out using dye-terminator chemistry (kit v.3, ABI 3130XL) and run on automated sequencer ABI Prism 3700 DNA Analyzer (*Applied Biosystems*). Reaction conditions and primer sequences are available upon request.

### *BGLAP* and *LMNA* genes relative expression

The relative expression level of both *BGLAP* and *LMNA* genes in blood was carried out in the proband and five controls by means of comparative DDCt method. Total RNA was extracted from whole blood with the use of PAXgene Blood RNA System (*PreAnalytiX*), according to standard protocols provided by the manufacturers. One microgram of total RNA was reversely transcribed using random hexamer primer (RevertAid First Strand cDNA Synthesis Kit; *ThermoFisher Scientific*), according to standard protocols provided by the manufacturers. Quantifications of the target and reference gene in the proband and controls was carried out using ViiA™ 7 Real-Time thermal cycler (*Applied Biosystems*). For each cDNA sample, all reactions were run in triplicate. Relative expression of *BGLAP* and *LMNA* in the proband was normalized to *TBP* reference transcript and to mean value of five controls. Reaction conditions and primer sequences are available upon request.

### Serum levels of bone turnover markers

Biochemical analyses of serum levels of alkaline phosphatase, inorganic phosphate, and total calcium were performed with the use of standard laboratory methods. The serum osteocalcin and intact parathyroid hormone levels were determined using chemiluminescence immunoassay (CLIA) (LIAISON XL; *Diasorin* and ADIVIA Centaur XP Immunoassay System; *Siemens* respectively). The results were compared to the laboratory reference ranges.

## Results

### Clinical report

**Fig. 1:**
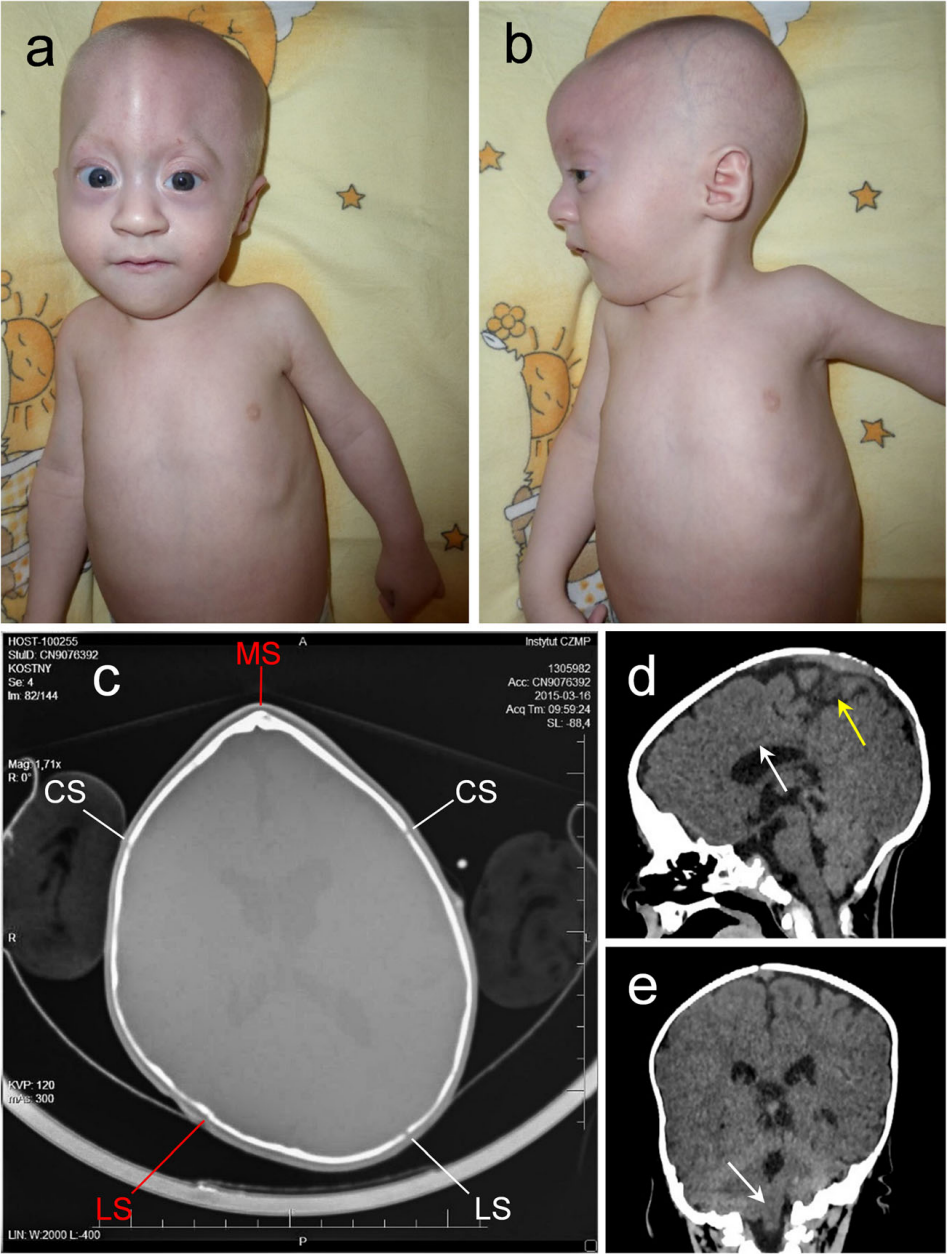
Facial view of the proband showing craniofacial abnormalities comprising **a** trigonocephaly with prominent metopic ridge, shallow orbits, proptosis, and hypotelorism and **b** flat facial profile, prominent occiput, and low-set, posteriorly rotated ears. **c** CT scan of the head showing premature synostosis of the metopic suture (trigonocephaly) and right lambdoid suture (indicated in red). MS—metopic suture, CS—coronal suture, LS—lambdoid suture. **d** CT scan of the head (sagittal view) showing thin and hypoplastic corpus callosum (marked with a white arrow) as well as morphological abnormalities of the median brain structures, including abnormal development of the white matter (marked with a yellow arrow). **e** CT scan of the head (coronal section) showing Chiari malformation; white arrow indicates downward displacement of the right cerebellar tonsil through the foramen magnum

**Fig. 2:**
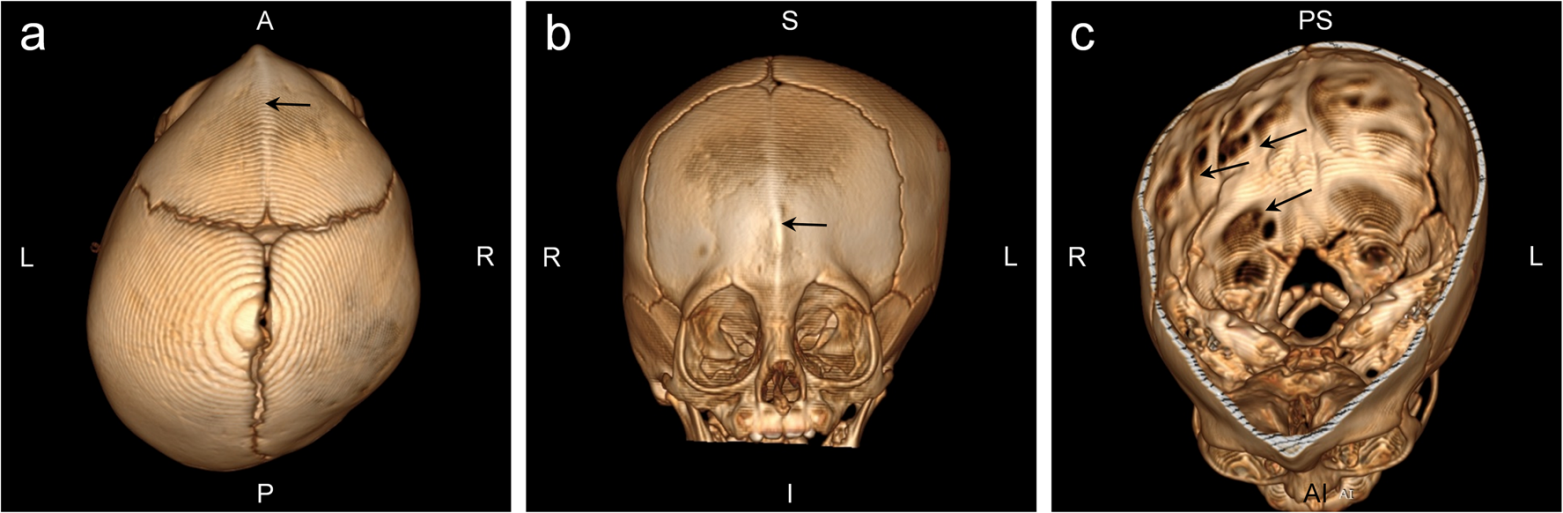
3D modeling of the patient’s skull. **a** Aerial view presenting asymmetric posterior plagiocephaly and prominent metopic ridge (black arrow) due to premature metopic synostosis. **b** Frontal view showing prominent metopic ridge (black arrow) and hypotelorism. **c** Horizontal section presenting prematurely closed right lambdoid suture with digitate impressions (indicated by black arrows) most marked in the right occipito-parietal region

The proband, a 5-month-old male patient of Polish ethnicity, was born by spontaneous delivery after uneventful pregnancy (G1P1) at 38 weeks of gestation to a non-consanguineous and healthy 32-year-old mother and a 33-year-old father. At birth, his weight was 2900 g (3rd–10th percentile), length 57 cm (75th–90th percentile), head circumference 30 cm (below 3rd percentile), and Apgar score was 10. Physical examination after birth showed trigonocephaly and associated facial dysmorphism. Abdominal and transfontanellar ultrasounds performed after birth were unremarkable. Hearing tests and ophthalmologic examinations were carried out during the first week after birth and repeated regularly throughout the first months of life and were normal. The boy was referred to the genetic clinic for diagnosis and first investigated by a clinical geneticist at the age of 5 months. Upon examination, he presented with global developmental delay, severe muscular hypotonia, and craniofacial dysmorphic features comprising prominent metopic ridge, trigonocephaly, high-arched palate, prominent occiput, hypotelorism, shallow orbits, and low-set, posteriorly rotated ears (Fig. 1a, b). His body weight was 5.5 kg (below 3rd percentile). CT scan of the head performed at the age of 7 months showed premature fusion of the metopic suture along with unilateral right-sided lambdoid craniosynostosis (Fig. 1c), as well as thin and hypoplastic corpus callosum, morphological abnormalities of the median brain structures, including abnormal development of the white matter (Fig. 1d), and Chiari malformation (Fig. 1e). 3D modeling of the patient’s skull performed with the use of RadiAnt DICOM Viewer showed posterior plagiocephaly (Fig. 2a) due to prematurely closed right lambdoid suture (Fig. 2c) and prominent metopic ridge due to premature metopic synostosis (Fig. 2a, b), hypotelorism (Fig. 2b), as well as digitate impressions most marked in the bone structures of the right occipito-parietal region (Fig. 2c). Upon neurological assessment at the age of 7 months, the patient presented with psychomotor retardation with global development that amounted to 3 months of age. Conventional chromosomal analysis performed on peripheral blood lymphocytes with a resolution of 550 bands per haploid genome showed normal male karyotype (46,XY).

### Serum levels of bone turnover markers

Osteocalcin serum level (224 ng/ml) was 3.4-fold higher as compared to the reference range (4.6–65.4 ng/ml). The serum concentrations of alkaline phosphatase (301 U/L), inorganic phosphate (4.6 mg/dl), and total calcium (10.7 mg/dl) were within normal ranges as compared to the reference values (142–335 U/L, 3.3–5.6 mg/dl, 8.8–10.8 mg/dl, respectively), while intact parathyroid hormone was slightly lowered (12.6 pg/ml) in comparison to the reference range (18.5–88.0 pg/ml).

### Array comparative genomic hybridization (array CGH)

**Fig. 3:**
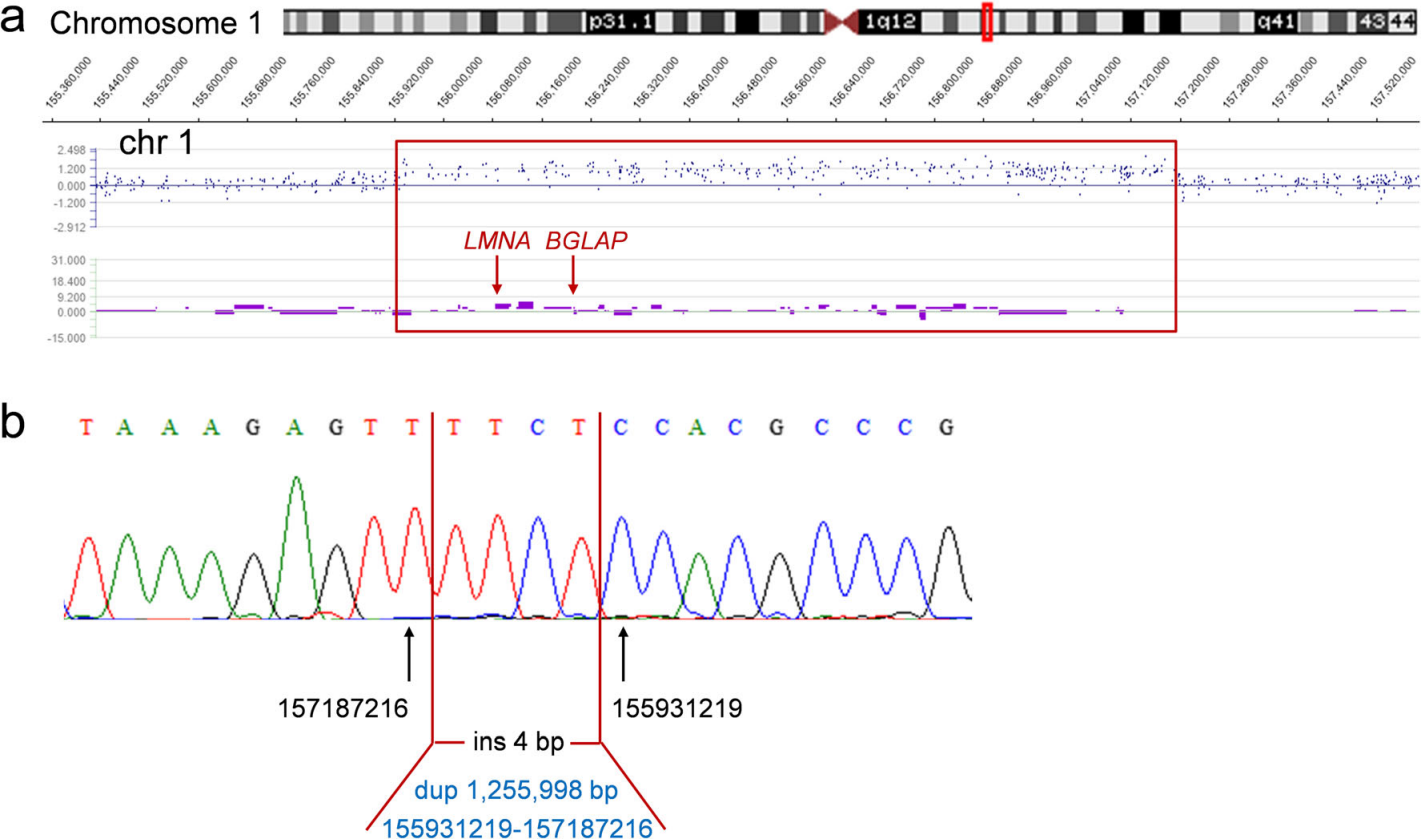
**a** Interstitial duplication in the index at 1q22-q23.1 encompassing *LMNA* and *BGLAP* genes, identified by means of array CGH (*Roche NimbleGen*). **b** Breakpoint sequencing results: a 1.26 Mb duplication (chr1:155931219–157187216; hg19), and an insertion of four nucleotides (TTCT) at one of the breakpoints

Array CGH detected a previously unreported duplication at chromosome 1q22-q23.1 (chr1:155927620–157198170; hg19), encompassing 42 protein coding genes, including *BGLAP*, encoding osteocalcin, and *LMNA*, encoding lamin A/C (Fig. 3a).

### Quantitative real-time PCR (qPCR) and breakpoint analysis

qPCR confirmed the 1q22-q23.1 duplication in the proband and excluded its presence in both unaffected parents. Subsequent rounds of qPCR narrowed down the region of duplication and allowed for the design of primers for breakpoint sequencing. Sequencing of the breakpoints revealed an insertion of four nucleotides (TTCT) at one of the breakpoints. The exact size of the duplication was 1,255,998 bp (chr1:155931219–157187216; hg19). Breakpoint sequencing results were shown in Fig. 3b.

### *BGLAP* and *LMNA* relative expression

**Fig. 4:**
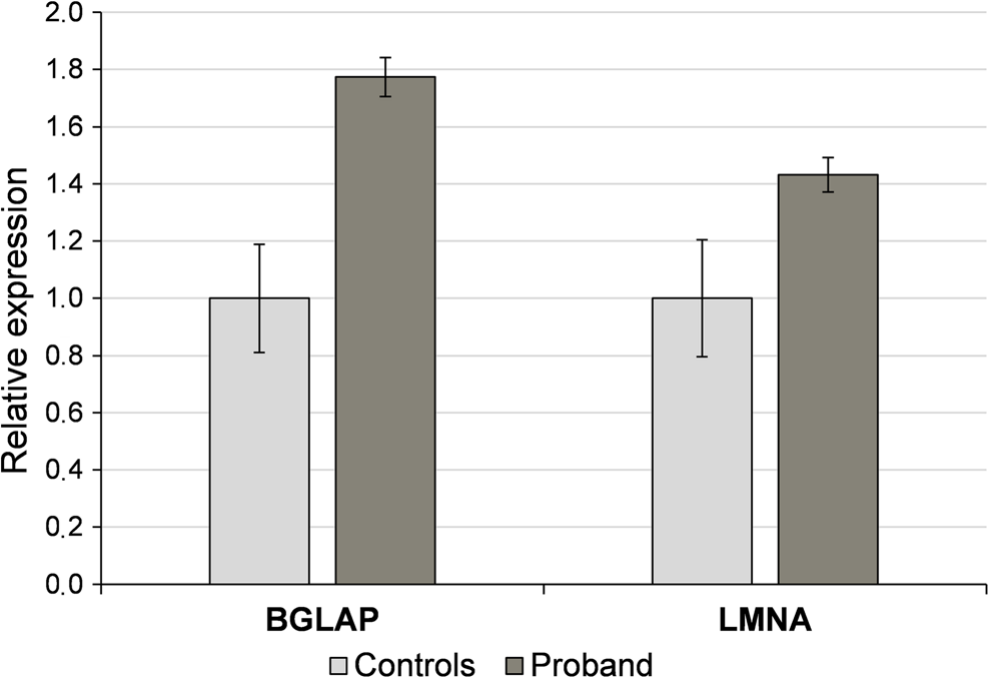
Relative expression levels of *BGLAP* and *LMNA* genes in blood samples of the proband and controls indicating 1.8-fold increase of *BGLAP* expression and 1.4-fold increase of *LMNA* expression in the proband compared to the mean value of five healthy controls. Error bars represent standard deviation

The analysis of *BGLAP* and *LMNA* relative expression performed in blood samples of the proband and five controls revealed 1.8-fold increase of *BGLAP* expression and 1.4-fold increase of *LMNA* expression in the proband in reference to the mean value of controls (Fig. 4).

## Discussion

Genetic changes underlying most of the CS types, especially other than coronal synostosis, remain largely unknown. A small proportion of metopic synostosis is caused by 9p22 deletions and point mutations in *FREM1* (Vissers et al. 2011). However, most of the cases remain undiagnosed at a molecular level. Complex CS may rarely result from mutations in a recently discovered gene *ERF*, which encodes for a transcription factor (Twigg et al. 2013). Furthermore, complex forms of the disease may be also caused by chromosomal rearrangements, including small submicroscopic segmental aberrations referred to as copy number variations (CNVs), which are estimated to account for 10–15% of CS (Stratton et al. 1986; Eshel et al. 2002; Wilkie et al. 2010; Massalska et al. 2014). Several interesting examples of CNVs resulting in craniosynostosis include *IHH* regulatory mutations (Klopocki et al. 2011), *RUNX2* duplications (Mefford et al. 2010), and *MSX2* duplications (Kariminejad et al. 2009). The fusion of cranial sutures is orchestrated by an interplay between collagen fibers and mesenchymal cells that differentiate into osteoblasts, osteoclasts and osteocytes upon mechanical stress. The molecular origin of CS has been linked to mutations in genes encoding osteoblastogenic proteins that trigger the premature osteogenesis in cranial sutures via different molecular pathways (Katsianou et al. 2016).

In this paper, we describe a previously unreported de novo 1.26 Mb duplication at chromosome 1q22-q23.1 identified in a sporadic male proband presenting with complex CS (metopic and right-sided lambdoid synostosis), muscular hypotonia, psychomotor retardation, and facial dysmorphism. The duplication encompasses 42 protein coding genes, including *BGLAP* and *LMNA*, both implicated in osteoblast differentiation, which are most probably involved in the pathogenesis of the clinical phenotype observed in our patient.

*BGLAP* encodes osteocalcin (OCN), the most abundant non-collagen component of bone extracellular matrix. The protein promotes matrix mineralization by high binding properties for calcium and hydroxyapatite, thus having a pivotal role in osteogenesis (Kruse and Kracht 1986; Sandberg et al. 1993; McGuigan et al. 2010). There are several signal transduction cascades involved in osteogenesis during cranial suture development, including the MAPK/ERK pathway (activated by FGFRs), SMAD signaling network, and Wnt/β-catenin pathway. Hyperactivation of these pathways results in premature closure of sutures leading to craniofacial abnormalities (Katsianou et al. 2016). Two major downstream target proteins for these signaling cascades, Runx2 and Msx2, are required as molecular switches for *Bglap* expression. The gene’s activity during osteogenesis reflects the stages of osteoblast differentiation. *Bglap* is transcriptionally repressed in proliferating cells by Msx2, whereas in mature osteoblasts Dlx3, Dlx5, and Runx2 are recruited to initiate the gene transcription. Thus, OCN is considered to be a major marker of mature osteoblasts (Hassan et al. 2004). Of note, studies on rat models demonstrated that *Bglap* expression level (as well as other bone-associated extracellular matrix molecules) is up-regulated in calvarial dura matter, directly underlying fusion of sutures (Greenwald et al. 2000). To date, genetic abnormalities directly affecting the *BGLAP* have not been reported to cause premature closure of sutures. However, several studies have determined how mutations in key elements of the aforementioned signal transduction pathways alter the *BGLAP* expression pattern in osteoblast derived from fused sutures. According to these reports, the gene expression was elevated in patients with Apert syndrome, as well as in mice harboring Fgfr1 Pro250Arg Pfeiffer syndrome mutation, thus confirming the direct correlation between the increased level of osteogenic proteins and the pathological ossification of sutures in both human and mice (Lemonnier et al. 2000, 2001; Zhou et al. 2000). These findings are consistent with the results of our study in which we observed an elevated blood expression of *BGLAP* and increased serum osteocalcin level in the proband, probably due to the identified duplication. Of note, the serum alkaline phosphatase (ALP) level was within a normal range. Based on our observations, we hypothesize that the CNV results in the general overexpression of *BGLAP* that may have occurred also locally during osteogenesis in fusing cranial sutures, leading to the craniosynostosis observed in our patient.

Another interesting gene included in the duplication found in our proband was *LMNA. LMNA* encodes lamin A/C, a nuclear intermediate filament protein that plays a major role in the structural organization of the nucleus and regulation of gene expression via interaction with signaling molecules and transcription factors (Aebi et al. 1986). The role of lamin A/C in osteoblastogenesis is associated with its potential to control the fate of mesenchymal stem cells (MSCs), which may differentiate into either osteoblast or adipocytes. Different mutations in *LMNA* disrupt the integrity of the nuclear envelope, mostly in the cells subjected to mechanical stress, leading to a group of disorders known as laminopathies (Manilal et al. 1999; Maraldi et al. 2006). Based on clinical features, laminopathies could be organized into four groups: muscle disorders, lipodystrophies, neuropathies, and accelerated aging disorders. The explanation of pleiotropic effect of various mutations in a single gene is attributed to the differences in the molecular mechanisms via which the variants affect the protein function. These include abnormal protein structure, alteration of its charge, or failure of the post-transcriptional processing of LMNA. Unprocessed protein is accumulated in the cells and incorporated into the nuclei where it interferes with the nuclear integrity, resulting in destabilization of the nucleus structure (Worman and Bonne 2007).

The pathomechanism underlying a variety of phenotypes found in human laminopathies has been investigated in both in vitro and in vivo studies. The features associated with *LMNA* loss-of function mutations arise from primarily affected MSCs and include reduction in muscle mass, defects of bone formation, and craniofacial symptoms, such as delayed closure of sutures as seen in mandibuloacral dysplasia (MAD) and Hutchinson-Gilford progeria (Novelli et al. 2002; De Sandre-Giovannoli et al. 2003; Shen et al. 2003; Yang et al. 2006; Kim et al. 2011). Studies using the spontaneous *Lmna* mutation (*Dhe*) mouse, which serves as a naturally occurring animal model for laminopathies, show that the cranial suture tissue that fails to fuse exhibits abnormal nuclear morphology, hypomineralization, and low level of collagen I and III expression (Odgren et al. 2010). Furthermore, the important role of *LMNA* in the formation of mineralized matrix has been demonstrated in vitro using human osteoblasts and MSCs. Lamin A/C knock-down in these cell lines resulted in impaired osteoblastogenesis, enhanced osteoclast formation, and increased adipogenesis, which was associated with reduced *RUNX2*, *BGLAP*, and *ALP* expression levels, as well as the impaired RUNX2 nuclear binding activity (Akter et al. 2009; Rauner et al. 2009). Consistent with these data, studies using *Lmna*^*−/−*^ mice showed decreased bone volume and muscular atrophy that occurred concomitantly with increased bone marrow and inter-myofiber fat infiltration, in the 4-week-old mutants as compared to controls (Tong et al. 2011). Since progressive muscle weakness is a principal sign in a number of laminopathies, including Emery-Dreifuss muscular dystrophy, limb-girdle muscular dystrophy, and Charcot-Marie-Tooth type 2B1 and has been reported in the *Lmna*^*−/−*^ mice, muscular hypotonia observed in our patient may also be attributed to the abnormal gene expression and functioning of *LMNA*. In addition, psychomotor retardation manifested by our proband may result not only from hypotonia and muscular weakness, but also from the impediment of the growth of the patient’s brain. This is often seen in complex forms of CS, even in cases where there is no overt evidence for increased intracranial pressure.

Interestingly, *LMNA* gene dosage may be also implicated in the pathogenesis of CS, as its overexpression correlates with an elevated level of osteogenic factors including RUNX2 and OCN (Bermeo et al. 2015; Tsukune et al. 2017). Consistently, we demonstrated that *LMNA* expression measured in peripheral blood of the index patient was elevated by 1.4-fold in comparison with healthy controls. This slight overexpression may contribute to craniosynostosis but is rather inconsistent with muscular hypotonia, as this feature is related to decreased level of *LMNA* (Tong et al. 2011). Considering a possibility that dosage imbalance of other than *LMNA* and *BGLAP* genes could contribute to the observed phenotype, we analyzed the predicted effect of haploinsufficiency (HI) of all protein-coding genes included in the duplication. Out of seven genes with a high (< 10%) or moderate (< 25%) HI score (*NES*, *CCT3*, *MEF2D*, *LAMTOR2*, *ETV3*, *SSR2*, *NTRK1*), none of them have been linked to craniosynostosis (Huang et al. 2010). A partly overlapping deletion in the 1q22-q23.1 region has been recently described in a patient presenting with intellectual disability and multiple congenital anomalies, and two of the HI genes, *NES* and *MAF2D*, were shown to be necessary for normal neuron development in mice (Ikeshima et al. 1995; Park et al. 2010; Aleksiuniene et al. 2018). Therefore, dosage imbalance could possibly contribute to mental retardation observed in the index patient.

In conclusion, we hypothesize that the complex phenotype of our proband, although caused be a novel 1q22-q23.1 duplication, does not result directly from the increased gene dosage of *LMNA* and *BGLAP*, but additionally from the change of a regulatory landscape within the duplicated region. As a consequence, misexpression of *LMNA* and *BGLAP* in a specific developmental time point during embryogenesis would give rise to the complex CS and muscular hypotonia observed in our patient, although further studies are needed to support this hypothesis.
