# Correction to: Novel 1q22-q23.1 duplication in a patient with lambdoid and metopic craniosynostosis, muscular hypotonia, and psychomotor retardation

**DOI:** 10.1007/s13353-018-0460-7

**Published:** 2018-08-25

**Authors:** Anna Sowińska-Seidler, Ewelina M. Olech, Magdalena Socha, Dawid Larysz, Aleksander Jamsheer

**Affiliations:** 1 Department of Medical Genetics, Poznan University of Medical Sciences, Rokietnicka 8 Street, 60-806 Poznan, Poland; 2 Department of Radiotherapy, The Maria Skłodowska Curie Memorial Cancer Centre and Institute of Oncology, Gliwice Branch, 44-101 Gliwice, Poland

**Correction to: Journal of Applied Genetics (2018) 59:281–289**

10.1007/s13353-018-0447-4

This article was originally published electronically on 29 May 2018 with incorrect copyright line in the Publisher’s internet portal (currently SpringerLink). The copyright line of the article should be “© The Author (s) 2018”.

